# Supplementary material for: Causal Relationship between Aging and Anorexia Nervosa: A White-Matter-Microstructure-Mediated Mendelian Randomization Analysis
Source: Biomedicines. 2024 Aug 16;12(8):1874. doi: 10.3390/biomedicines12081874 (PMC11351342; doi:10.3390/biomedicines12081874)
Supplement: Supplementary file 1 [file biomedicines-12-01874-s001.zip › Supplementary Figure/Figure S4/Singe SNP plot of MR analysis for TL to ubm-b-2104.pdf]

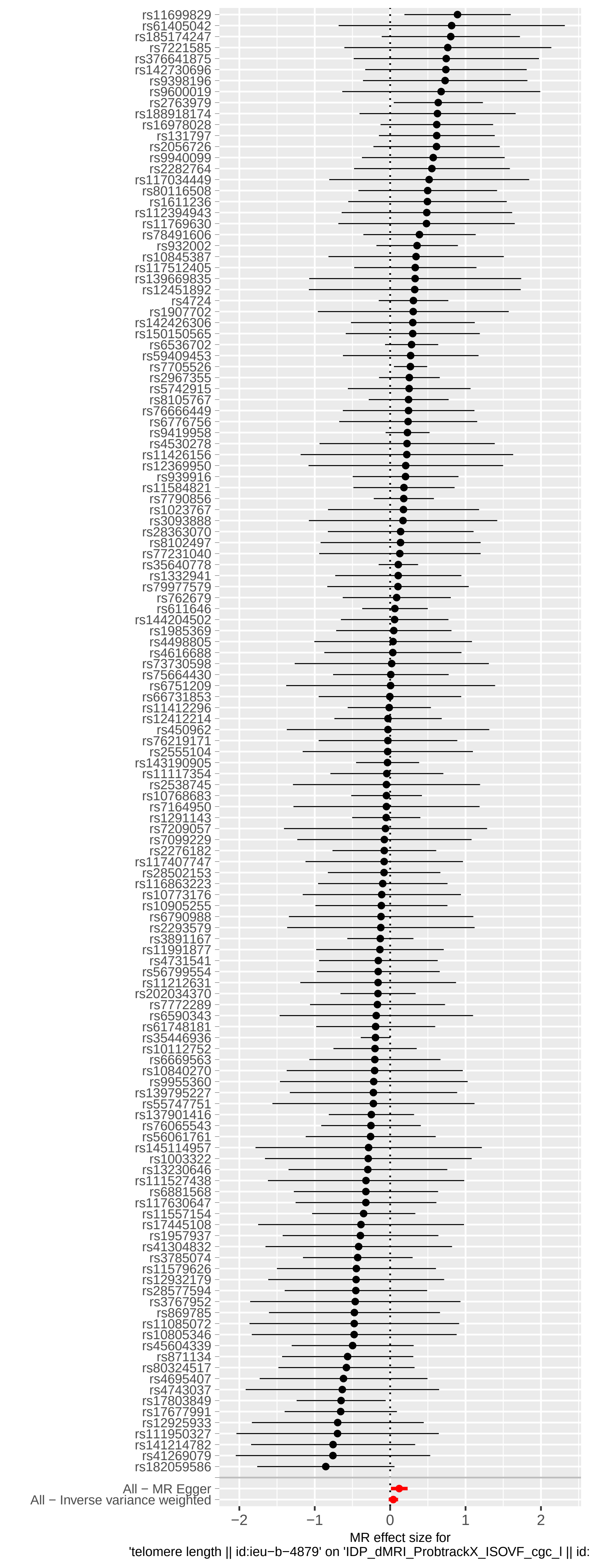

rs11699829
rs61405042
rs185174247
rs7221585
rs376641875
rs142730696
rs9398196
rs9600019
rs2763979
rs188918174
rs16978028
rs131797
rs2056726
rs9940099
rs2282764
rs117034449
rs80116508
rs1611236
rs112394943
rs11769630
rs78491606
rs932002
rs10845387
rs117512405
rs139669835
rs12451892
rs4724
rs1907702
rs142426306
rs150150565
rs6536702
rs59409453
rs7705526
rs2967355
rs5742915
rs8105767
rs76666449
rs6776756
rs9419958
rs4530278
rs11426156
rs12369950
rs939916
rs11584821
rs7790856
rs1023767
rs3093888
rs28363070
rs8102497
rs77231040
rs35640778
rs1332941
rs79977579
rs762679
rs611646
rs144204502
rs1985369
rs4498805
rs4616688
rs73730598
rs75664430
rs6751209
rs66731853
rs11412296
rs12412214
rs450962
rs76219171
rs2555104
rs143190905
rs11117354
rs2538745
rs10768683
rs7164950
rs1291143
rs7209057
rs7099229
rs2276182
rs117407747
rs28502153
rs116863223
rs10773176
rs10905255
rs6790988
rs2293579
rs3891167
rs11991877
rs4731541
rs56799554
rs11212631
rs202034370
rs7772289
rs6590343
rs61748181
rs35446936
rs10112752
rs6669563
rs10840270
rs9955360
rs139795227
rs55747751
rs137901416
rs76065543
rs56061761
rs145114957
rs1003322
rs13230646
rs111527438
rs6881568
rs117630647
rs11557154
rs17445108
rs1957937
rs41304832
rs3785074
rs11579626
rs12932179
rs28577594
rs3767952
rs869785
rs11085072
rs10805346
rs45604339
rs871134
rs80324517
rs4695407
rs4743037
rs17803849
rs17677991
rs12925933
rs111950327
rs141214782
rs41269079
rs182059586
All − MR Egger
All − Inverse variance weighted
−2
−1
0
1
2
MR effect size for
'telomere length || id:ieu−b−4879' on 'IDP_dMRI_ProbtrackX_ISOVF_cgc_l || id:
